# α-Spectrin regulates cell shape changes during disassembly of microtubule-driven protrusions in *Drosophila* wings

**DOI:** 10.17912/micropub.biology.001169

**Published:** 2024-04-15

**Authors:** Ngan Vi Tran, Martti P. Montanari, Dmitri Lubenets, Léa Louise Fischbach, Hanna Antson, Yasushi Okada, Yukitaka Ishimoto, Tambet Tõnissoo, Osamu Shimmi

**Affiliations:** 1 Institute of Molecular and Cell Biology, University of Tartu, Tartu, Estonia; 2 Institute of Biotechnology, University of Helsinki, Helsinki, Uusimaa, Finland; 3 The University of Tokyo, Tokyo, Japan; 4 RIKEN, Wako, Saitama, Japan; 5 Akita Prefectural University, Akita, Japan

## Abstract

The dynamics of microtubule-mediated protrusions, termed Interplanar Amida Network (IPAN) in *Drosophila* pupal wing, involve cell shape changes. The molecular mechanisms underlying these processes are yet to be fully understood. This study delineates the stages of cell shape alterations during the disassembly of microtubule protrusions and underscores the pivotal role of α-Spectrin in driving these changes by regulating both the microtubule and actomyosin networks. Our findings also demonstrate that α-Spectrin is required for the apical relaxation of wing epithelia during protrusion disassembly, indicating its substantial contribution to the robustness of 3D tissue morphogenesis.

**Figure 1. α-Spectrin regulates IPAN-mediated cell shape changes:**
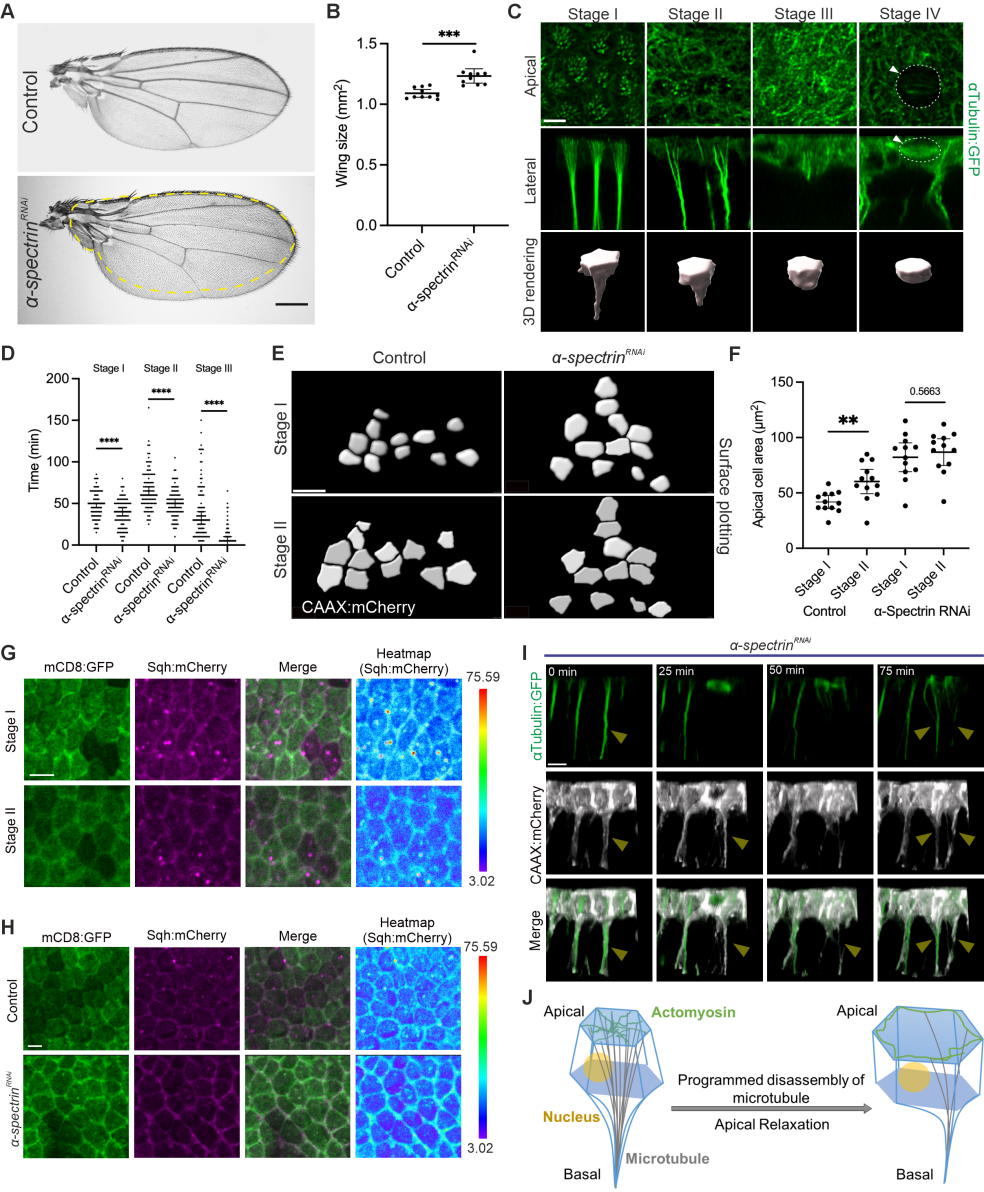
**A.** Adult male wings in control (upper panel) and in conditional RNAi knockdown of *α-spectrin* (lower panel). Yellow dotted line represents the size of the control wing. **B.** Adult male wing size in control and conditional RNAi knockdown of *α-spectrin* . ***P = 0.0001. Data are means ± 95 % confidence intervals (CIs). Statistical significance was calculated by two-tailed t-test. **C.** Four stages in cells in which microtubule protrusions regress, with subsequent mitosis. Stage I: individual microtubules have clear apical foci (top row, apical view), and join to form a microtubule cable further basally (middle row, lateral view). Cell shapes are shown by 3D rendering (bottom row). Stage II: Fewer individual microtubule foci are found apically, and basal microtubule cable structure is less focused. Stage III: microtubules no longer form apical foci, and the microtubule cable has disassembled. Stage IV: A microtubule spindle and mitotic cell rounding have been observed as the cell undergoes mitosis (white arrowheads). **D.** Graphs showing distribution of cells in Stages I, II and III within the region of interest (ROI) in control and conditional RNAi knockdown of *α-spectrin* . Data are means ± 95 % CIs. Each data point represents one cell. ****P < 0.0001. Data were analyzed by two-sided Mann-Whitney U test. **E, F.** Surface plots (E) and quantification (F) of apical cell area segmented by CAAX:mCherry (white) in control (left) and conditional RNAi knockdown of *α-spectrin* (right). Stage I (upper) and stage II (lower) cells were selected at 10.5h after puparium formation (APF) and 12.5h APF (29ºC), respectively. Control cells undergo apical relaxation during progression from Stage I to II, whereas *α-spectrin* RNAi knockdown cells do not show apical relaxation. **P = 0.0037. Data are means ± 95 % CIs. Statistical significance was calculated by two-tailed t-test. **G, H.** Apical view of mCD8:GFP (first column), Sqh:mCherry (second column) and merged images (third column) of stage I (upper) and stage II (lower) pupal wing epithelial cells (G). Comparison of Sqh:mCherry signal intensity between control (upper) and conditional RNAi knockdown of *α-spectrin* (lower) (H). Heat map showing intensities of Sqh:mCherry signal (fourth column). A scale for the heat maps is indicated on the right. The heat map was produced using a spectrum heat map function with Imaris 9.7. **I.** Cells reform microtubule protrusions after cell division in *α-spectrin* RNAi knockdown. A lateral view of αTubulin:GFP (green) and CAAX:Cherry (white) in the pupa wing epithelium is shown, captured every 25 minutes. The yellow arrows indicate a cell undergoing mitosis, which then reforms its protrusion after division. **J.** Schematics of IPAN-mediated cell shape change from stage I to stage II, involving the disassembly of microtubule protrusions and apical relaxation regulated by α-Spectrin. Created with BioRender.com. Scale bars: 250µm (A), 5µm (C, G, H, I), 10µm (E).

## Description

Wing development in *Drosophila* is a classical model for elucidating genetic regulation of tissue morphogenesis. The larval wing imaginal disc, a monolayer epithelium, is widely utilized to study the integration of diverse signaling molecules in tissue growth and pattern formation (Tripathi and Irvine, 2022) . During metamorphosis, this monolayer transforms into a bilayered primordial wing, comprising dorsal and ventral epithelia. The developmental process within the first 24 hours after puparium formation (APF) unfolds into three distinct phases. Initially, the wing disc evolves into a bilayered structure during the first phase (0-10h APF, first apposition). In the subsequent phase (10-20h APF, inflation), the two epithelia separate and later re-appose during the third phase (at 20h APF, second apposition) (Fristrom et al., 1993; Gui et al., 2019; Montanari et al., 2022) . Notably, cell proliferation in the pupal wing primarily occurs during the inflation stage (Etournay et al., 2016; Milan et al., 1996) . Our latest research highlights the critical function of a microtubule-mediated protrusion network, which we have termed IPAN in driving proliferation within 3D epithelial tissues (Tran et al., 2024) . The IPAN sustains the connection between the two-layered epithelia of the inflated pupal wing and then loses cell-cell contacts through the degeneration of microtubules, leading to coordinated mitoses.

To understand the molecular mechanisms underlying pupal wing development, we carried out *in vivo* RNAi screening using a pupal wing-specific approach. We selected candidate genes based on the adult wing phenotypes that exhibited larger or smaller sizes than the control, or showed inflated structures. Knockdown of *α-spectrin* revealed it one of the candidate genes that resulted in a larger adult wing size. Spectrin proteins function with multiple interacting partners, including microtubules and actin filaments (Deng et al., 2020; Fletcher et al., 2015; Forest et al., 2018; Khanal et al., 2016) . When *α-spectrin* was conditionally knocked down in wing epithelia using the *nub-Gal4* driver (Forest et al., 2018) , an increase in wing tissue size was observed ( Fig. 1A, B ). Therefore, we hypothesize that α-Spectrin may be critical in regulating IPAN-mediated proliferation.

Since the dynamics of microtubule protrusions involve cell shape changes (Ko et al., 2019; Matis, 2020; Plochocka et al., 2021; Singh et al., 2018) , we clarify the changes in cellular structures over time and designate the following stage numbers ( Fig. 1C ):

*Stage I* : The majority of αTubulin is utilized for membrane protrusions, with ~30 microtubule foci per cell found medio-apically (Tran et al., 2024) .

*Stage II* : Vertical microtubule projections are partially degenerated, and the number of microtubule foci decreases to 10 or fewer per cell.

*Stage III* : Vertical microtubule projections are mostly degenerated; accordingly, basal protrusions are lost, and a lateral fiber of microtubules is observed medio-apically and cortically.

*Stage IV* : αTubulin is utilized to form the mitotic spindle, and mitotic cell rounding occurs (dotted line) (Gibson et al., 2006) .

To understand the time-dependent alterations in cells, we selected 25 cells from time-lapse imaging, repeated across four independent datasets, and analysed the cellular stages. We measured the duration of stages I through III by tracking individual cells ( Fig. 1D ). We then analyzed the cellular stages of conditional knockdown in *α-spectrin ^RNAi^* wings. We found that the degeneration of stage I microtubule protrusions occurred faster in *α-spectrin ^RNAi^* wings than in control wings. When comparing the lifetimes of stages II and III, both were significantly shorter in *α-spectrin ^RNAi^* wings than in control wings ( Fig. 1D ). These data suggest that α-Spectrin-mediated cell shape changes are one of the means to regulate IPAN-mediated proliferation by controlling the temporal sequence of gradual changes in microtubule structures from vertical protrusions to mitotic spindles.

Interestingly, we found that apical cell size fluctuates during cell shape changes while microtubule protrusions are disassembled. When we measured the apical cell area by tracking individual cells between stage I and stage II ( Fig. 1E ), we observed that the apical area is smaller in stage I and then increases in stage II, a process termed apical relaxation ( Fig. 1E, F ) (Godard et al., 2020) . When α-Spectrin was conditionally knocked down, apical areas are larger size than in control in stage I, and do not show significant expansion from stage I to II ( Fig. 1E, F ). Since it has been argued that medioapical Myosin II localization is tightly linked to the apical constriction of epithelial cells (Booth et al., 2014; Gillard et al., 2021; Le and Chung, 2021; Toddie-Moore et al., 2022) , we imaged live membrane-bound mCD8:GFP and Spaghetti squash (Sqh):mCherry (myosin II light chain)-expressing pupal wings in stage I and II. We found that substantial amounts of Sqh:mCherry were observed medioapically and cortically in stage I. Most Sqh:mCherry was then detected cortically, but not medioapically, in stage II, corroborating apically constricted cells relaxing from stage I to stage II ( Fig. 1G ). We further attempted to investigate the spatial distribution of Sqh:mCherry in *α-spectrin* knockdown tissue ( Fig. 1H ). Our data suggest that α-Spectrin is required for apical relaxation between stage I and stage II, likely mediated through the regulation of MyoII localization.

Moreover, while observing time-lapse imaging of αTubulin:GFP and CAAX:mCherry in both control and *α-spectrin ^RNAi^* wings, we noticed that the dynamics of cell shapes are affected in *α-spectrin ^RNAi^* wings. When microtubule protrusions are disassembled, membrane protrusions are also disassembled simultaneously in control tissue. In contrast, membrane protrusions are occasionally observed even after microtubule protrusion disassembly and the mitotic phase in *α-spectrin ^RNAi^* wings ( Fig. 1I ). Strikingly, microtubule protrusions are often regenerated after mitosis if membrane protrusions remained intact ( Fig. 1I ).

These results indicate that α-Spectrin plays an important role in regulating cell shape changes related to the timing of stages of the IPAN structure and the robustness of IPAN structure dynamics. Therefore, α-Spectrin is a crucial factor in supporting tissue homeostasis during IPAN-mediated morphogenesis (Fig 1J).

## Methods

***Drosophila* genetics**

Crosses were set up and maintained at room temperature (~22ºC) to generate pupal wings for imaging RNAi knockdown transgene expression phenotypes. Pupae from crosses with RNAi transgene-bearing flies were transferred to 29⁰C 16 hours before collecting white pupae. These white pupae were then kept at 29⁰C until imaging commenced at 10.5h after puparium formation (APF) (equivalent to 13.5h APF at 25⁰C). Head eversion served as a developmental marker to assess the suitability of pupae for live imaging. Male pupae were selected for imaging due to their slightly smaller size, and to maximize αTubulin:GFP expression, benefiting from dosage compensation (the *ubi>αTubulin:GFP* transgene is located on the X-chromosome).

**Full genotypes**

Figure 1A, B :

Control: *w, ubi>αTubulin:GFP; nub-Gal4, ubi>cnn:RFP /+; tub>Gal80 ^ts^ /+*

*α-spectrin* ^RNAi^ : *w, ubi>αTubulin:GFP; nub-Gal4, ubi>cnn:RFP/+; tub>Gal80 ^ts^ / UAS-α-spectrin ^RNAi^*

Figure 1C : *w, ubi>αTubulin:G* FP (X)

Figure 1D :

Control: *w, ubi>αTubulin:GFP; ubi>cnn:RFP/+; tub>Gal80 ^ts^ /+*

*α-spectrin ^RNAi^* : *w, ubi>αTubulin:GFP; nub-Gal4, ubi>cnn:RFP/UAS-α-spectrin ^RNAi^ ; tub>Gal80 ^ts^ /+*

Figure 1E, F :

Control: *w, ubi>αTubulin:GFP; nub-Gal4>UAS-CAAX:mCherry/+; tub>Gal80 ^ts^ /+*

*α-spectrin* ^RNAi^ : *w, ubi>αTubulin:GFP; nub-Gal4>UAS-CAAX:mCherry/UAS-α-spectrin ^RNAi^ ; tub>Gal80 ^ts^ /+*

Figure 1G : *w; ap-Gal4>UAS-mCD8:GFP/+; tub>Gal80 ^ts^ / Sqh:mCherry*

Figure 1H

Control: *w; ap-Gal4>UAS-mCD8:GFP/+; tub>Gal80 ^ts^ / Sqh:mCherry*

*α-spectrin* ^RNAi^ : *w; ap-Gal4>UAS-mCD8:GFP/ UAS-α-spectrin ^RNAi^ ; tub>Gal80 ^ts^ / Sqh:mCherry*

Figure 1I : *w, ubi>αTubulin:GFP; nub-Gal4>UAS-CAAX:mCherry/UAS-α-spectrin ^RNAi^ ; tub>Gal80 ^ts^ /+*

**Preparing pupal wings for live imaging & Live imaging of pupal wings**

The live imaging protocol has been described previously (Tran et al., 2024) .

The live imaging process was performed using a Leica TCS SP8 STED 3X CW 3D. The raw confocal data was saved as .lif files and later converted to .ims files using Imaris File Converter from Oxford Instruments. All the subsequent steps were completed in Imaris from Oxford Instruments.

**Quantifying cell distribution across stages**

Samples were live-imaged at 5-minute intervals for four hours (10.5h - 14.5h APF at 29°C) as described previously (Tran et al., 2024) . Cell distribution analysis was performed using αTubulin:GFP data to classify stage I, stage II, stage III, and stage IV in 25 random individual cells per wing. The experiment was repeated four times.

**Quantifying apical cell areas**

To visualize the apical cell region, CAAX:mCherry was expressed under the control of *nub-Gal4* in both control and *α-spectrin* RNAi. Samples were live-imaged in for two hours (10.5h - 12.5h APF at 29°C). To analyze apical cell areas in control and *α-spectrin* RNAi samples, the apical surfaces of individual cells in the ROI region were measured during stage I and stage II.

## Reagents

**Table:** 

STRAIN	GENOTYPE	AVAILABLE FROM
*Drosophila melanogaster*	*ap-GAL4*	Bloomington Drosophila Stock Center (BDSC) #3041
*Drosophila melanogaster*	*nub-GAL4*	BDSC #25754
*Drosophila melanogaster*	*UAS-α-spectrin RNAi*	BDSC #42801 (Fig.1 A, B) BDSC #56932 ( Fig. 1D -F, H, I)
*Drosophila melanogaster*	*w;; tub>Gal80ts*	BDSC #7017
*Drosophila melanogaster*	*sqh:mCherry*	BDSC #59024
*Drosophila melanogaster*	*UAS-CAAX:mCherry*	BDSC #59021
*Drosophila melanogaster*	*UAS-mCD8:GFP*	BDSC #32186
*Drosophila melanogaster*	*w, ubi>αTubulin:GFP*	From C. Gonzalez (Rebollo et al., 2004)
